# Federated causal discovery in medicine: trends, opportunities, and challenges

**DOI:** 10.3389/fdgth.2026.1846020

**Published:** 2026-07-17

**Authors:** Niccolò Rocchi, Marco Scutari, Alessio Zanga, Radha Nagarajan, Fabio Stella

**Affiliations:** 1 Department of Informatics, Systems and Communication (DISCo), University of Milano-Bicocca, Milan, Italy; 2 Department of Epidemiology and Data Science, Fondazione IRCCS Istituto Nazionale dei Tumori, Milan, Italy; 3 Istituto Dalle Molle di Studi sull’Intelligenza Artificiale (IDSIA), Lugano, Switzerland; 4 Rady Children’s Health, Orange, CA, United States

**Keywords:** causal graph, federated architecture, federated learning, healthcare, medicine

## Abstract

Exponential growth and continued digitisation have accelerated the adoption of data-driven and evidence-based approaches in medicine. This includes deciphering associations, including potential causal associations, from multivariate observational biomedical data under certain implicit assumptions. Such an evidence approach marks the shift from classical hypothesis testing to discovery and hypothesis generation. Widespread adoption of common data models has especially accelerated collaborative approaches in medicine while transitioning from centralised to federated architectures that facilitate discovery without the explicit sharing of sensitive medical data. This perspective provides an overview of causal discovery from observational data with a focus on federated learning. Specifically, it outlines the trends, opportunities, and challenges of federated causal discovery in medicine. While medical research has traditionally relied on the hierarchy of evidence generated from the evidence pyramid, the ability of federated causal discovery to facilitate evidence generation collaboratively from heterogeneous sources is expected to enhance the generalizability and transportability of findings while addressing sample size considerations—a critical aspect for its successful and widespread adoption in medicine.

## 1 Introduction

During the last decade, an exponential growth in *multivariate* and *multimodal* biomedical data has taken place due to several factors, including the widespread adoption of digital technology, continued digitisation efforts across a multitude of source systems, and high-throughput assays (1). While multivariate data typically consists of *relational* and *structured* data that can be represented in a tabular format (e.g., numerical and categorical features), multimodal data includes *non-relational* and *unstructured* data (e.g., texts and images). Multivariate data are typically stored in *data warehouses* for querying and downstream analytics. *Centralized* and *federated* warehouse architectures have been proposed for the same (2). A brief description of these architectures, along with their pros and cons, is shown in Table 1.

**Table 1:** Pros and cons of centralised and federated architectures.

Architecture	Pros	Cons
Centralised	Centralised governance with representation from the participating institutions.	A centralised resource is a single point of failure.
	Single storage and compute infrastructure for querying and analytics.	Centralising high-throughput data may have prohibitive bandwidth and storage costs.
	Compute architecture is straightforward compared to federated ones.	Varying compliance, lack of trust, and regulatory regimes across institutions may limit data access and the analyses permitted.
Federated	Architectures are more robust to failures because of distribution.	Architectures are more complex and difficult to manage.
	No explicit data sharing, enhanced trust across the participating institutions, and reduced patient consent burden.	Data drift and uneven data distributions across institutions can impact training and the generalizability of models.
	More control across the participating institutions. Data are compliant with local governance.	Execution of queries and analytics can have relatively higher latencies in federated architectures.

There is an increasing interest in harnessing information from multivariate and multimodal observational data to improve patient outcomes in an evidence-based manner. This includes modelling associations, including causal associations, from multivariate observational biomedical data, complementing traditional evidence generation (3). Such a data-driven approach, combined with domain knowledge, has the potential to validate established associations and discover novel ones for critical assessment, thereby generating new hypotheses and research questions. It also has the potential to provide novel system-level insights, a precursor to the development of targeted interventions, tailored treatment regimens, and disease management strategies (4). More importantly, it can complement the hierarchy of evidence from the *evidence pyramid*, including systematic reviews and *randomised controlled trials* (RCTs), which are widely used in medicine (3).

Networks have proven to be especially useful abstractions in this regard (5), with *nodes* representing the variables of interest, and *edges* their associations. These networks can be modelled across multiple scales (e.g., molecular, clinical, or demographic data) of varying granularity and resolution (6), as well as from cross-sectional and longitudinal profiles. While longitudinal profiles capture the explicit temporal evolution of multivariate processes, they are usually challenging to generate from economic and stationarity standpoints. The latter requires controlling several factors to preserve statistical properties over time (7). Not surprisingly, cross-sectional profiles that interrogate the multivariate process within a chosen time window, in conjunction with replicate measurements, have been prevalent. While earlier attempts focused on modelling these networks by estimating pairwise dependencies between variables [e.g., relevance networks (8)], there is increasing evidence that dependence between a pair of variables need not be direct, warranting the inclusion of conditional dependencies. Techniques such as *causal Bayesian networks* (CBNs) (9) have proven particularly helpful in this regard, with broad application in medicine and healthcare. These include deciphering associations from sequencing (10), molecular (11), epidemiological (12) and electronic health records (13) with a focus on oncology (14), neurology (15), rare (16) and infectious diseases (17), among others. CBNs have also been used successfully to model relationships from diverse data sets, including multi-centre (18), temporal (19), state-space (20), partially observed (21), and multimodal data (22) under the broad theme of *causal discovery* (CD) (23). We refer to Supplementary Section S1, for further details.

This perspective examines applications of *federated causal discovery* (FCD) in medicine and healthcare. Specifically, it introduces and describes the “*What*”, “*Why*”, and “*How*” of causal networks, while offering a comprehensive and critical overview of FCD, including its opportunities and challenges. The manuscript does not cover core components of causal inference in the usual medical and methodological sense, such as explicit estimands, identification strategies, adjustment logic, estimation procedures, or sensitivity analysis (24, 25). A detailed description of causal representation, causal discovery, and federated learning is provided in the Supplementary Sections S1 and S2 for completeness. Definitions and popular algorithms for FCD are discussed in Section 2. Section 3 describes the study that motivated us to write this paper, which is used to illustrate a practical application of FCD. Section 4 presents current trends, opportunities (4.1), and challenges (4.2) of FCD with a focus on medicine.

## 2 Federated causal discovery

*Federated causal discovery* (FCD) extends classical CD to multiple data sources without explicit data sharing. In this sense, it aims to find the structure of one (or more) CBNs that explain the data-generating mechanism behind the measured data. A single global CBN is most appropriate when the underlying data-generating process is assumed to be identical across all participating sites, such as in pooled homogeneous populations. In contrast, allowing the CBN to vary can capture site- or patient-specific causal mechanisms is more appropriate when there are differences in demographics, clinical protocols, or disease subtypes. An FL taxonomy is shown in Table 2, and described in Supplementary Section S2. FCD is a subset of FL. In particular:

- FCD is typically implemented on horizontally partitioned data, as it is challenging to compute sufficient statistics involving variables that are never observed jointly across clients. Typically, it cannot identify the true *causal graph* (CG) because multiple *directed acyclic graphs* (DAGs) may equally explain the data at hand (26).
- FCD algorithms follow an offline learning procedure, as online CD is typically challenging even in single-source settings. This is not a significant limitation, as clinical data are more often collected retrospectively than prospectively.
- There are instances of multi-task FCD in the literature, such as FMT-NBN (27). They typically assume a shared CG (Section 2.1) to be realistic. How to account for distribution shifts among clients remains an open research question (Section 4.2).
- Synchronous aggregation is commonly carried out, although asynchronous learning may be practicable in principle.
- Most privacy-preserving techniques in FCD involve sharing scores or statistics, which are sometimes encrypted to protect the data. The extent to which original data can actually be traced back from scores or statistics remains an unexplored area (Section 4.2).
- CD requires significant computational resources and time for big data, which favours the cross-silo architecture. There are no examples of (fully) distributed FCD, but it could be a solution to develop personalised models for multi-task FL.

FCD often makes additional assumptions, which are discussed in the following section.

**Table 2:** FL taxonomy based on different traits.

Trait	Type	Description
Partitioning	Horizontal Vertical Hybrid	Full overlap in variables, no overlap in data points. No overlap in variables, full overlap in data points. Partial overlap in variables, partial overlap in data points.
Learning	Offline Online	Model is learnt once from the available data. Model is updated whenever new data are available.
Task	Single-task Multi-task	Learning one global model. Learning different models for different clients.
Aggregation	Synchronous Asynchronous	Each client contributes to each aggregation. Some clients may not contribute to each aggregation.
Sharing	Model Knowledge Synthetic data	The entire local models are shared with the server. Model’s intermediate information is shared. Local synthetic data are shared instead of raw data.
Scale	Cross-silo Cross-device	Few clients, high computational power, and availability. Many clients, limited computational power, and availability.
Topology	Star schema Distributed	One server is in charge of aggregation. Multiple aggregators are allowed.

Yellow represents the data partitioning schema, blue represents the algorithmic structure and task, and red represents the client-server architecture. Underlined terms identify the framework of FCD within FL; exceptions are marked in Supplementary Figure S2.

### 2.1 Assumptions

In the most general setting, a source-dependent *structural causal model* (SCM) is assumed to be responsible for generating each local data set $D^{1},\ldots ,D^{k}$. Specifically, let $M^{1},\ldots ,M^{K}$ be a collection of SCMs underlying the K data sources where $M^{k}$ is given by:

$$\begin{array}{rl} M^{k} & =(U^{k},X^{k},F^{k},P^{k}(U^{k})),\\ X_{i}^{k} & \;:=\;f_{i}^{k}(\Pi_{i}^{k},U_{i}^{k}). \end{array}$$

Without loss of generality,^1^ we set $U^{k}=U,\forall k$. Because FCD research and applications focus overwhelmingly on the horizontally-partitioned data, we set $X^{k}=X,\forall k$.

**Figure 1:**
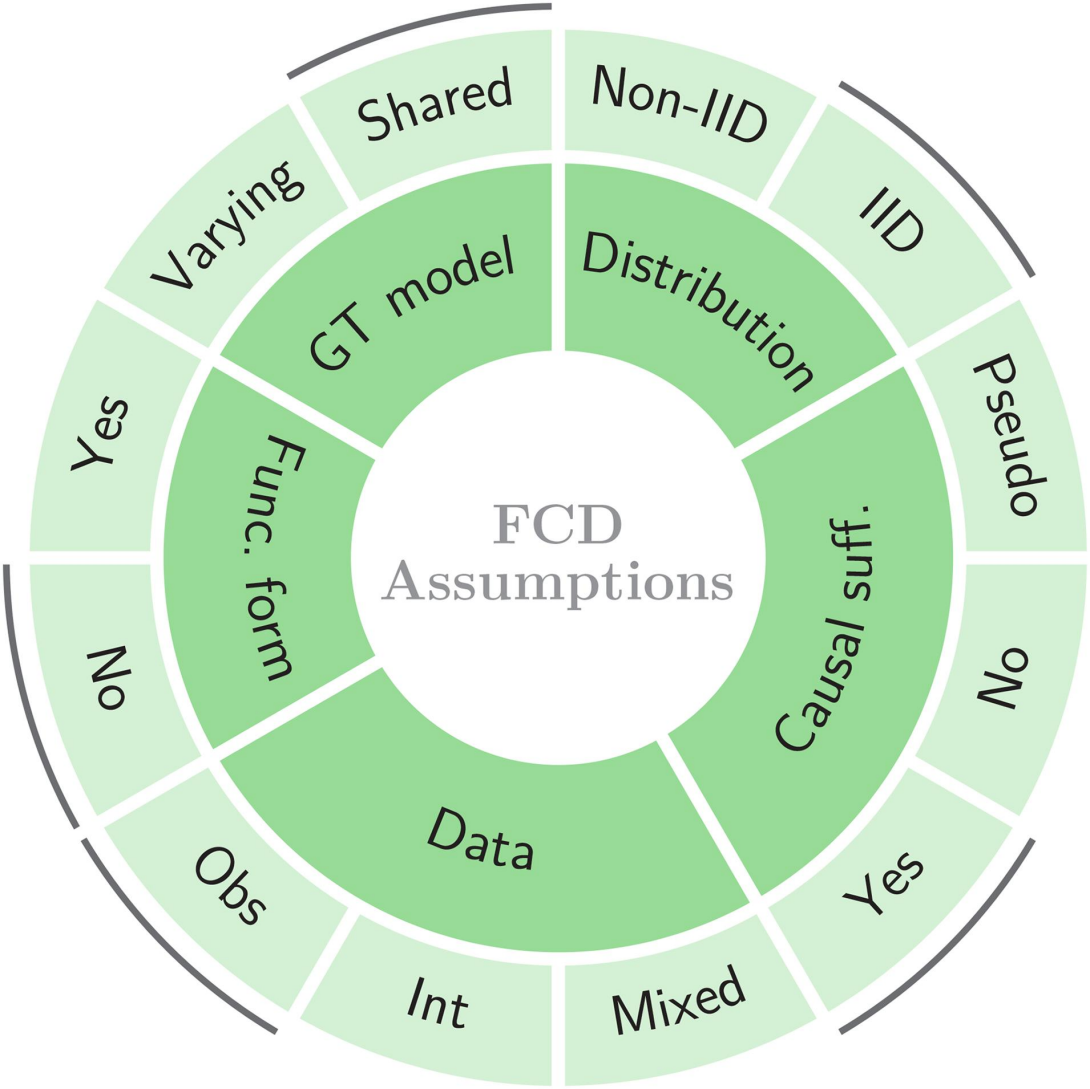
Overview of possible assumptions in FCD. Gray small tabs identify the most adopted ones; exceptions are marked in Figure 2, Supplementary Materials. “GT model” stands for “ground-truth model”, namely the CG; “Func. form” means “functional form” and identifies whether the approach needs to specify the functional dependencies in the SCMs; “CS” stands for causal sufficiency.

Figure 1 lists a set of common assumptions regarding both the model and the data. They are relevant beyond FL, but we discuss them in the context of FCD.

Assumption 1 **Shared causal graph.**

Cause-and-effect relationships are invariant across clients, that is $\Pi_{i}^{k}=\Pi_{i},\forall i,k$. Hence, all CGs coincide: $G^{k}=G,\forall k$.

In real medical systems, measurement protocols, treatment policies, patient populations, and institutional workflows may differ substantially across sites. Therefore, Assumption 1 may not always be realistic, as in the case of fMRI data (28). Even when the cause-and-effect relationships are population-invariant, the networks’ parameters may vary (29). For instance, disease aetiology and drug mechanisms of action are similar regardless of where a physician collects measurements, but drug effectiveness may vary across subpopulations. For a more in-depth discussion, we refer the reader to Section 4.

Under Assumption 1, FCD consists of obtaining a single G from $D^{1},\ldots ,D^{k}$. Otherwise, we talk about *multi-task* FCD as the task of collaboratively finding $G^{k},\forall k$.

Assumption 2 **Observational data.**

Data were collected during routine work without deliberate manipulations of the variables. In other words, the variables are not *intervened* on (9).

For a client k and variable $X_{i}$, a *perfect* intervention sets a structural assignment for $X_{i}$ to $X_{i}\;:=\;x_{i}$ for a certain $x_{i}$. On the other hand, an *imperfect* intervention modifies either $P^{k}(U)$ or $f_{i}^{k}(\cdot )$ in $M^{k}$ without fixing them to a single, deterministic value.

Assumption 3 **Pseudo-causal sufficiency.**

Any variable causing two or more variables in $X^{k}$ is included in $X^{k},\;\forall k$.

Assumption 3 coincides with the standard *causal sufficiency* assumption holding locally at each data source (30). However, multi-source causal sufficiency requires stronger guarantees, as one variable may cause two other variables measured at different sites.

Assumption 4 **Causal sufficiency.**

Any variable causing two or more variables in $\underset{k}{⋃}X^{k}$ is included in $X^{k},\;\forall k$.

The hybrid and vertical data partitioning settings conflict with Assumptions 3 and 4 because not all variables are necessarily measured across all sources. Note that causal sufficiency implies pseudo-causal sufficiency, but not vice versa. Unmeasured confounding, selection bias and measurement errors can all be seen as different instances of violations of causal sufficiency, and they can lead to incorrect causal edges even when CD runs in a federated setting. An illustrative example contrasting Assumptions 3 and 4 will be presented in Section 3.

Assumption 5 **IID data.**

Data are *independent and identically distributed* (IID) within and across the clients, hence $P^{k}(U)=P(U),\forall k$.

**Figure 2:**
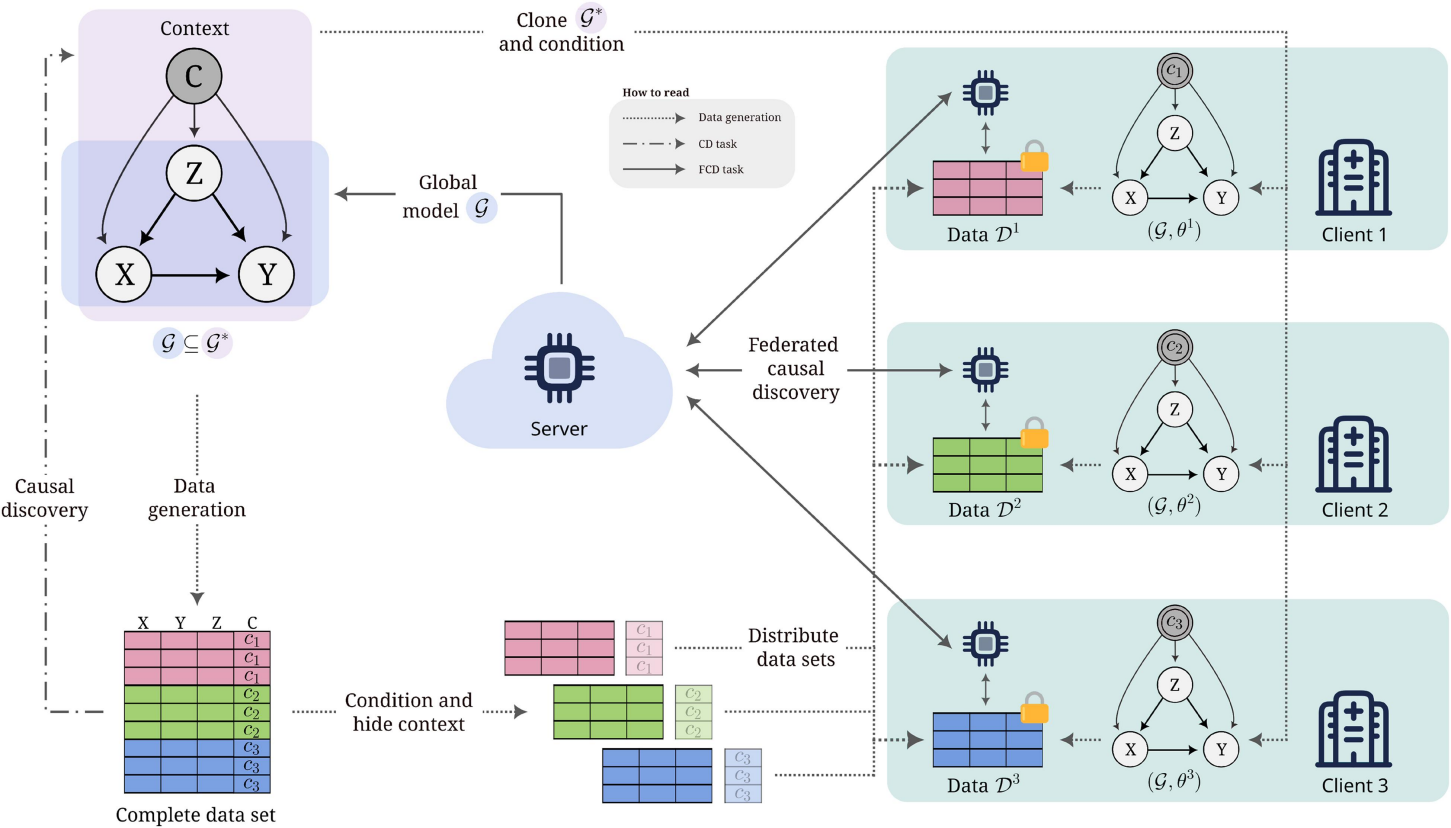
Overview of data generation in the federated setting, along with the CD and FCD tasks. Note that C may represent multiple variables.

Non-IID data commonly arise from *distribution drifts*, or *shifts*, between different populations: $P^{i}(X)\neq P^{j}(X)$, for some i≠j. If Assumptions 2 and 3 hold, we can account for them using a set of *context variables* as illustrated in Figure 2 (31). Consider the CBN $\left(G^{*},\theta^{*}\right)$, where (i) $G^{*}$ is the enhanced version of G obtained by adding a set of variables C and an edge from C∈C to X∈X whenever $P^{i}(X)\neq P^{j}(X),\;i\neq j$; (ii) $\theta^{*}$ parametrizes the joint distribution $P^{*}(X,C)$. Each assignment C=c defines specific environmental, individual, or measurement conditions (31). The local data set $D^{k}$ is collected under the environment $C=c^{k}$, and the underlying CBN is $\left(G,\theta^{k}\right)$. Here, $\theta^{k}$ parametrizes the joint distribution $P^{k}(X)=P^{*}(X,C|C=c^{k})$.

Hypothetically, $G^{*}$ can be learned whenever the context C is recorded at each client. However, those variables are usually unknown, so at least one indicator variable C can serve as a proxy, with C=k denoting the k-th client (2). Note that this approach requires the server to know which client each local update originates from, which is not always the case in highly private settings.

### 2.2 Algorithms

Causal discovery using multiple data sets is well-explored in the literature (31). Basic approaches to FCD involve performing CD locally and aggregating the learned graphs through edge voting, union, or intersection (32). These approaches prove effective when local sample sizes in individual clients are large enough and Assumption 5 assumption holds. However, smaller sample sizes at the client level (Figure 2) may not be representative of the underlying population, leading to statistically significant differences in local distributions. Moreover, Assumption 5 may not be satisfied in many scenarios.

Table 3 presents the main FCD algorithms linked to the assumptions and settings discussed in Section 2.1.^2^ Each works under horizontal data partitioning, except for FMT-NBN, which can be applied to non-horizontal settings. The table columns and notable algorithmic instances are detailed in the caption.

**Table 3:** Characterisation of the main FCD algorithms.

		Method			Assumptions			Application			
#	Name	Class	Share	Acycl.	Suff.	Int.	SCM	Data	RWD	Scal.	Code
Wang et al. (36)	FedC${}^{2}$SL	Constr.	Stat.	✓				D	✓	(100, 10), (50, 64)	✓
Li et al. (37)	FedCDH	Constr.	Stat.	✓	✓(p)			C, D	✓	(30, 10), (6, 32)	✓
Mian et al. (34)	PERI	Score (D)	Score	✓	✓			C (∗)	✓	(15,9), (9, 100)	✓
Mian et al. (38)	RFCD	Score (D)	Score	✓	✓			C (∗)		(10, 7)	
Yang et al. (39)	FedCausal	Score (C)	Param. (subset)	✓	✓			C	✓	(80, 10)	
Liu et al. (40)	NOTEARS-PFL	Score (C)	Param.		✓		✓	C	✓	(80, 10), (50, 64)	
Ng and Zhang (41)	NOTEARS-ADMM	Score (C)	Param.		✓			C	✓	(100, 10), (50, 64)	✓
Liu et al. (42)	FedCASL	Score (C)	Param.		✓			C	✓	(40, 10), (30, 32)	
Ye et al. (43)	DARLS	Average	Param., grad.	✓	✓		✓	C, D	✓	(70, 20)	✓
Huang et al. (44)	FedPC	Average	Struct.	✓	✓			C, D	✓	(441, 15)	✓
Gao et al. (33)	FedDAG	Average	Param.		✓		✓	C	✓	(40, 10)	✓
Qiu and Yang (45)	Bloom	Average	Param.		✓	✓	✓	C	✓	(100, -)	
Abyaneh et al. (46)	FedCDI	Average	Struct.		✓	✓		D	✓	(20, 8), (37, 4)	✓
Guo et al. (47)	FedECD	Average	Struct.		✓			D (∗)		(27, 30)	
Guo et al. (48)	FedCSL	Average	Substruct.		✓			D (∗)	✓	(5000, 12)	✓
Guo et al. (49)	FedACD	Average	Struct.		✓			C, D	✓	(37, 20)	✓
Yu et al. (50)	FedLCS	Average	Struct.		✓			C, D	✓	(400, 15)	
Yang et al. (27)	FMT-NBN	Average	Param.		✓		✓	C	✓	(20, 4)	
Torrijos et al. (51)	FedGES	Ensemble	Struct.	✓	✓			D (∗)		(724, 100)	✓

**Method.** **Class:** “Constr.” (constraint-based method), “Score” (score-based, discrete (“D”) or continuous (“C”) score), “Average” (with a voting strategy server-side) or “Ensemble” (complex model fusion). **Share:** information sent to the central server, summary statistics (“stat.”), model score (“Score”), parameters (“Param.”), gradients (“grad.”) or the DAG structure (“Struct.”). The DAG could have edge weights (FedCSL) or an assigned belief (FedCDI). **Acycl.:** whether the algorithmic output is a DAG or requires additional processing.

**Assumptions.** **Suff.:** whether the causal or pseudo-causal (“p”) sufficiency assumption is required. **Int.:** methods that can exploit interventional data. **SCM:** whether assumptions about the variables’ functional assignments are needed (such as linearity).

**Application.** **Data:** handles discrete (“D”) or continuous (“C”) data; a (∗) symbol is present whenever the extension to another data type is feasible, for instance, by changing local sufficient statistics. No method is available for mixed discrete/continuous data, but extensions may be possible. **RWD:** whether experiments on real-world data have been carried out. No method has been tested on survival or longitudinal data, but NOTEARS-PFL and FedDAG have been on imaging data sets. **Scalability:** the maximum number of (nodes, clients) on which the method has been tested.

Except for FedC${}^{2}$SL, in which clients share encoded statistics, no other method directly addresses data privacy. Almost all of them argue that data leakage is prevented by claiming that no client data are exposed. While Gao et al. (33) acknowledge that avoiding data sharing is only a partial solution, the extent to which the model’s parameters or structure reveal sensitive information remains unclear in the literature. As acknowledged by Mian et al. (34), differential privacy (35) can be achieved by injecting a certain amount of noise into the broadcast from client to server. Moreover, techniques such as secure multi-party computation (36) and encryption could be exploited and revealed to be effective in other domains. However, the feasibility of these approaches in FCD, the computational burden they entail, and their implications for privacy protection are underexplored (see Section 4.2).

#### 2.2.1 Constraint

The FedC${}^{2}$SL algorithm (36) extends PC and the *fast causal inference* (FCI) algorithms (23) to the federated setting. FCI works in contexts where some variables may be unobserved, while PC assumes causal sufficiency (Assumption 3). The server iteratively constrains the DAG space using *federated conditional independence tests* that distribute the computation of sufficient statistics and securely aggregate them. The FedCDH algorithm (37) does the same, but clients release sufficient statistics to the server only once.

#### 2.2.2 Score

The PERI algorithm (34) builds on the Greedy Equivalent Search (GES) algorithm (52). It minimises the worst-case *regret* of a function that measures how far a DAG is from the DAG that best fits the data across all clients. PERI ensures privacy and convergence guarantees. The FedCausal algorithm (39) extends the gradient NOTEARS-ADMM algorithm (41) by providing a global optimisation function.

#### 2.2.3 Ensemble

Model ensemble techniques consist of finding a *consensus* DAG from a given collection (53). The FedGES algorithm (51) uses model ensemble techniques to combine locally learned DAGs at the server level. The procedure is iterative: the global DAG is fused with the client’s DAG, becoming the starting point for the subsequent local optimisation. Note that naive aggregation of edges, such as union or averaging, does not ensure acyclicity, while edges lose their causal and statistical meanings. The FedLCS algorithm (50) is a local FL extension of the PC algorithm that finds only the edges incident on a single variable, rather than the entire CG.

## 3 A motivating example

Limited data availability frequently constrains cancer research, as do lengthy ethical approval processes and stringent requirements for protecting patient privacy. These challenges substantially hinder the development and validation of data-driven methodologies in real-world clinical settings, and motivate our use of FCD.

### 3.1 The clinical problem

Endometrial cancer (EC) is a cancer of the endometrium of the uterus. Approximately 90,000 patients die each year due to EC, calling for more research on personalised EC treatments (54). In this context, pelvic and para-aortic lymph node metastases (LNM) are among the most important prognostic factors for choosing adjuvant treatment and improving survival in node-positive EC. Approximately 10% of endometrial cancer patients present with LNMs at diagnosis according to clinical literature (54). The clinical problem is therefore to disentangle the interplay between administered treatments, LNM status, and other covariates to support clinicians in choosing the optimal therapy for each patient to maximise the survival and to reduce the risk of relapse (55).

### 3.2 The distributed data setting and why centralisation was not feasible

The European Network for Individualised Treatment of Endometrial Cancer (ENITEC) study and the PIpelle prospective ENDOmetrial carcinoma (PIPENDO) study (55) involved 19 gynaecological oncology clinics. Each clinic collected detailed data on patient demographics, histopathological features, administered treatments, and outcomes. Centralising these multi-institutional patient records in a single repository for analysis was not feasible due to stringent privacy regulations, lengthy ethical approval processes, and institutional data governance policies governing sensitive oncological data.

### 3.3 The choice of FCD method

Cancer research data often contain missing values, and the reasons for these missing values may not be random but rather informative about how data are collected. Zanga et al. (55) developed the first FCD algorithm that accounts for local missing-data distributions. In particular, the authors extended existing CD methods for missing data to the federated setting, relaxing the underlying assumption of a global missing mechanism because the missingness patterns in each clinic’s data differed, reflecting local clinical workflows, referral patterns, or documentation practices. Failing to model federated missingness could bias the learned causal structure. While such an approach could be implemented using any of the methods mentioned in Section 2.2, score-based CD was the most convenient from a mathematical perspective.

### 3.4 How clinicians validated the learned causal graph

Involving clinicians was a vital part of this knowledge elicitation process: experts provided a set of causal and probabilistic statements that the CBN had to satisfy. Firstly, the model variables were selected based on which factors the clinical experts considered relevant for predicting survival and the presence of LNMs (56, 57). Secondly, the CBN’s causal pathways were validated against prior clinical knowledge through systematic literature reviews to corroborate the causal claims implied by the graph (55). Clinicians could also validate the CBN’s predicted probabilities for specific events against the statements they provided to assess its calibration.

### 3.5 Downstream clinical decision support

The learned CBN offers new insights into how treatments interact with the observed variables. This directly supports clinical decision-making by helping clinicians reason about the likely effect of alternative adjuvant treatments on LNM progression and patient survival for specific patient profiles, thereby moving toward a more personalised treatment strategy. FCD produced a CBN that generalised significantly better and achieved higher causal accuracy than those derived from single-source studies, consistent with other landmark studies using non-causal models (58–61). Furthermore, an overall sensitivity analysis showed that FCD also improved model robustness (55).

### 3.6 Contrasting pseudo-causal and causal sufficiency

Each of the 19 clinics collected the same set of patient variables, such as tumour grade, lymph node status, and treatment. Pseudo-causal sufficiency implies that within any single clinic’s data set, there are no unmeasured common causes that jointly affect the recorded variables, even though clinics might differ in unobserved factors, such as local care pathways, that do not directly confound the measured relationships. In contrast, causal sufficiency would require that if a variable, such as a specific genetic mutation, caused, say, the choice of adjuvant therapy at one clinic and the survival outcome at another, that mutation would need to be measured and included at all 19 sites. This stronger condition is often impractical in federated healthcare settings where each institution may collect a slightly different set of covariates. Yet, pseudo-causal sufficiency still permits each clinic’s local causal structure to be learned without requiring that every possible confounder is shared or recorded across the entire network.

## 4 Discussion

As noted earlier, increasing digitisation in medicine has led to an exponential growth in multivariate and multimodal observational data. Deciphering novel patterns and associations from these data sets has the potential to discover novel associations while validating what is known. However, non-causal machine learning approaches focus exclusively on predictive performance and are not designed for clinical reasoning (62).

FCD has the potential to decipher causal patterns from these observational data sets across, without explicit data sharing, overcoming sample size constraints. Beyond providing transparent and mechanistic views of observed systems, CBNs obtained through FCD provide the ground for *causal inference* (Supplementary Section S1.2). Under specific assumptions (63) such as correct measurement, model faithfulness, and the absence of unmeasured confounding (ensured by Assumption 4), CBNs may yield valid conclusions about treatment or exposure effects and risk-factor modification, with the potential to ultimately improve patient outcomes. Moreover, they may reduce the risk of mistaking correlation patterns for causation, thereby providing a principled approach to investigating scenarios in which RCTs are infeasible and bridging the gap between observational and experimental evidence.

Still, they require large, well-balanced samples to produce transferable models that generalise across clinical and population cohorts and between different clinical cohorts. Crucially, the close relationship between several key concepts in causal inference and trial design (for instance, backdoor adjustment vs. randomisation, collider bias and stratification, path analysis and mediation) makes CBNs a powerful tool for boosting systematic reviews and RCTs.

In the remainder of this section, we will focus on opportunities and challenges of FCD in this context, as well as less-explored research areas.

### 4.1 Opportunities

Building on the effectiveness of FL (58, 59) and CD (62), FCD leverages decentralised *real-world evidence* (RWE) to ultimately improve drug discovery and patient outcomes, as observed above. By also pooling information from external control arms with real-world control patients from different institutions, the learned CBNs may be exploited to improve trial effectiveness, fairness, and data integration (64).

#### 4.1.1 Clinical translation

The need for cross-institutional cooperation to foster medical research has been apparent for years. Experts’ collaboration and data sharing enhance RWE, thereby strengthening the generalizability of analyses, reducing local biases, and promoting fairness (65). Popular architectures enabling multi-institutional storage and analytics of medical data include both centralised and federated architectures (Table 1).

Centralised architectures have been adopted to accelerate precision medicine research efforts, such as the *All of Us* initiative funded by the National Institutes of Health (NIH) (66). All of Us aims to provide equitable access to diverse data sets from participating institutions in a secure, centralised cloud environment, with centralised governance and a paid model to support analytics. Similarly, the European ELIXIR infrastructure supports the coordination and development of life sciences research, including a network of cross-domain experts, and promotes best practices for data analysis (67). The *Health Information Exchange* (HIE) also aims to improve care coordination while supporting surveillance (68). In addition, the *Global Alliance for Genomics and Health* (GA4GH) (69) aims to establish standards for sharing genomic and health data while ensuring privacy, security, and adherence to ethical values.

Unlike centralised architectures, federated architectures support querying of de-identified medical data, with the potential to accelerate clinical trials. An example is the *Shared Health Research Information Network* (SHRINE), funded by the *National Center for Advancing Translational Science* (NCATS) (70). SHRINE has been adopted by *Patient Centered Outcomes Research* (PCORI), enabling federated querying of healthcare data from PCORnet members. While these established projects targeted cohort discovery, new developments are focusing on model learning through FL instead.

FL has gained traction in health research, with studies exploring different data types and applications (65). To this end, several initiatives promoted the creation of distributed infrastructures to host ready-to-use, private data. Among those, the *IDEA4RC* project focuses on creating a decentralised ecosystem of rare cancers data in Europe, compliant with the standardisation of common data models (71); *FeederNet* is a South Korean initiative supporting the development of a bio-health data ecosystem for federated analyses (72). In principle, these platforms could be leveraged for large-scale FCD.

However, limited applied research on FCD has been published to date. Zanga *et al.* (55) evaluated their FCD algorithm on decentralised data sets related to endometrial cancer. Their method is suited to incomplete data, especially when the missing mechanism differs across centres. Chen *et al.* (73) modelled gene expression by leveraging a time-dependent version of the CG. Zhang *et al.* (74) proposed a method to aggregate outputs from different CD algorithms and applied it to the treatment of acute kidney injury. Other applied works (75, 76) focused on *federated causal inference* by assuming the CGs are known instead of learning them. Furthermore, no existing distributed medical data set currently provides a ground-truth DAG for FCD benchmarking and experimentation.

Cross-institutional collaborations may aim to learn more robust CBNs through FCD than individual institutions can; the reference population may be clearly specified and taken into account (see Section 4.2.2) (77). Involving their respective experts in the CD process increases the trust in the learned CBN and fosters its adoption in clinical practice. However, it may present challenges in harmonising heterogeneous and potentially conflicting domain knowledge (78).

#### 4.1.2 Efficiencies in RCTs

RCTs are limited in their ability to translate to real-world scenarios, such as cancer care, due to their inclusion and exclusion criteria (79). Non-causal models have shown mixed performance in providing information comparable to that from RCTs from RWE available in electronic health records (80). CBNs, on the other hand, can effectively harness RWE to better understand the real-world patient experience and outcomes thanks to their ability to learn digital twins of such settings (13). CBNs can identify relevant hypotheses to test and subpopulations with different treatment responses; provide prior estimates of effect sizes to identify relevant biomarkers for drug development (81); perform power analysis for sample size determination; and reduce the reuse of trial control arms, which limits transferability (82). In addition, they could be used more often to answer secondary analyses of existing RCT data (83).

Furthermore, CBNs can inform RCT design and assess its feasibility using observational RWE by emulating it within the “target trial” framework. Making sound design decisions about the length of follow-up, sample size, potential confounders, relevant subpopulations, minimisation of lost-to-follow-up, and expected cost of treatment delivery is essential for trials to provide useful information at their conclusion (84). At the same time, RCTs are increasingly expensive, reaching a median budget of $650,000, of which 27.4% is spent in the planning phase and 12.7% in the finalisation phase (85). Even so, about 70% of RCTs exceeded the budget by over 50%. Furthermore, 57% of RCTs had one or more substantial amendments, each costing $141,000 to $535,000 (86). Of these, 45% originated from protocol design flaws, inconsistencies in the protocol narrative, and infeasible eligibility criteria, which were “avoidable” and would have been flagged by a “target trial” CBN built from readily available RWE. Any additional planning efficiencies and reduced overruns resulting from more targeted designs are essential to advancing evidence-based healthcare in a cost-effective manner.

#### 4.1.3 Fairness

Fairness is a well-documented issue in clinical trials. In addition to biases in historical control data (87), they may also be biased due to limitations in the trial design (88), self-selection among minorities (89), physicians’ implicit biases (90), and other factors. FCD offers a comprehensive solution to this class of issues because of its unique combination of FL and causal inference.

FL can potentially provide better coverage of a target population by allowing multiple institutions to pool information without sharing patient-level data, which is one of the major concerns that pushes minorities to self-select themselves out of trial enrollment. Indirectly, it would also reduce the impact of physicians’ implicit biases by giving them access to scrutinised, larger control arms (91).

In addition to mitigating known sources of biases, better population coverage provides CD with the data it needs to construct a CBN that captures the characteristics of the trial and its patients. Such a CBN serves as the foundation for achieving *counterfactual fairness* (92), which builds on counterfactuals to examine differential outcomes as a function of legally protected attributes. This is the most rigorous framework for fairness assessment and remediation in the literature; notably, it goes beyond descriptive statistics and allows for disambiguation between biases mediated by distinct causal pathways (93).

#### 4.1.4 Synthetic data generation

Artificial intelligence models require large amounts of patient health data to build healthcare systems that improve diagnosis and understanding of disease. Issues with patient privacy and limited data availability make synthetic data generation an attractive solution to train those models (94).

CBNs are increasingly explored as generative models for synthetic data generation (95), with successful applications using the UK Clinical Practice Research Datalink (96). FCD can, at the same time, provide larger data sets to train more realistic CBNs for this purpose and serve as a reproducible data source for studying the performance, biases and systematic distortions of FCD algorithms. There is a strong research interest in investigating how a CBN, learned from multiple data sources and thus susceptible to various biases and limitations, can or must be used to generate reliable data.

### 4.2 Challenges

#### 4.2.1 Data heterogeneity

Most clinical data are stored in heterogeneous data silos with different logical and physical structures, each one tailored to meet specific technical needs. Combining different databases may lead to inappropriate and biased results, even within the same institution (97).

A software solution to data harmonisation is given by *Beacon v2* (98), which provides a secure and flexible protocol for querying heterogeneous databases. However, Beacon does not support FL. Collaborative networks such as *Observational Health Data Sciences and Informatics* (OHDSI) (72) provide *common data models* (CDMs) to address data heterogeneity in medicine, thereby improving the generalizability of findings. *Common* refers to the data logical structure and shared vocabularies, fostering homogeneous semantics. A CDM enhances data owners’ management, improves data users’ interoperability while complying with privacy and security standards, and facilitates the development of standardised analytical tools. The *Observational Medical Outcomes Partnerships* (OMOP) CDM (99), developed within OHDSI, provides a longitudinal view of each patient. Its *oncology extension* further increases the information granularity to best support cancer research.

Translation into CDMs via *extract-transform-load* (ETL) processes poses several challenges. Only a subset of a centre’s raw data is typically mapped, and ineffective cooperation among centres will impact the final data homogeneity. A lack of ETL expertise can also undermine the final data quality, leading to spurious cause-and-effect relationships in FCD. Both issues must be considered in FCD, and the ETL process must be well-documented for this purpose. Methodological research should also focus on developing FCD techniques for longitudinal and censored data (100), as well as modalities such as medical imaging (101) and natural language processing (64).

#### 4.2.2 Distribution and semantic drifts

Variable distributions may differ between sources due to genetic population structure, heterogeneous environmental conditions, including unmeasured economic and social factors, and other covariates, such as age and sex (102). The observational or interventional nature of various parts of the data is also a difficult assumption to test. Heterogeneous machine calibration, both across centres and over time, differing institutional policies, and various inductive and deductive processes employed by physicians, may effectively amount to unobservable interventions. Note that interventions differ from distribution drift across populations, as the latter holds irrespective of whether an intervention has been performed (103). Also, there may be *semantic drift* when variables have different semantics across data sources.

Modelling incomplete data poses similar challenges. The reason behind the presence of missing values is inherently causal; each data source in FCD may have not only varying degrees of missingness but also different missingness mechanisms. Expert prior knowledge and specialised algorithms are required to handle them (55). Discarding incomplete observations or assuming an identical missingness mechanism will bias both ETL and FCD itself.

#### 4.2.3 Aggregation bias

Learning CBNs from a single data source is a well-explored problem; the same is not true in FCD. Under Assumption 1, one may average all the local distributions, weighting them by the local sample size. However, this would not account for distribution drifts across sources. Even a single low-quality or highly biased data set can disproportionately bias the global CBN when few clients are present or when the data are class-imbalanced. Druzdzel and Díez (77) demonstrated how not combining knowledge from different sources or using only data from the setting in which the CBN will be used is neither necessary nor sufficient to ensure model correctness. Hence, whether to adopt FL depends on the population(s) of interest, the research question, and the validity of assumptions. A sensible approach could be to perform a posteriori global model personalisation for individual clients, especially when the research question pertains to the local population. However, determining the value of each client’s contribution to FCD is an open research question.

#### 4.2.4 Privacy and security

FCD algorithms must implement privacy and security by design to fulfil legal and ethical regulations, considering that institutions may be located in different jurisdictions (104). Privacy involves safeguarding and controlling personal information, while security means protecting the system’s integrity, availability, and confidentiality. The nature of attacks against privacy (e.g., *membership inference*) and security (e.g., *model poisoning*) depends on the threat model and the attacker’s goal (105, 106). Adversaries can be both internal and external, but a defence-in-depth strategy that couples FL’s architecture with cybersecurity best practices and encryption can render many such attacks impractical with current technologies (107).

Privacy and security should be addressed on multiple fronts (64). Membership inference attacks aim to determine whether a specific individual’s data was included in the training set, model poisoning attacks maliciously manipulate training data or model updates to corrupt the learned model, and model inversion attacks seek to reconstruct sensitive training data from model outputs or parameters. Both can originate from participating parties as well as external adversaries.

Cybersecurity best practices will prevent the most common data leaks. FL is designed to avoid data sharing, thereby preserving privacy by design; most privacy attacks in the literature are only feasible under unrealistic assumptions (108). Indeed, FL’s privacy-by-design approach aligns with the organisational measures required by GDPR (Articles 24, 25, and 32) and thereby facilitates compliance with both GDPR and the EU AI Act (107). Secure multi-party computation (36) (MPC, for encryption) and differential privacy (35) (DP, for privacy) can reach an acceptable trade-off between privacy, performance, and fairness (105). However, MPC incurs substantial computational overhead, and DP may degrade diagnostic or prognostic accuracy. Research to better understand the optimal cost-benefit application of these techniques is ongoing (109, 110). Notably, this privacy–accuracy trade-off is orthogonal to the federated learning framework; it is instead specific to the type of causal model being federated, as demonstrated by differentially private causal discovery algorithms that exhibit similar accuracy losses even in centralised settings (105, 111).

The scarcity of existing literature limits a comprehensive analysis of privacy and security in FCD. Membership inference attacks are not possible by sharing an unparameterised CG, but the presence or absence of specific edges might reveal sensitive information about certain subpopulations (112). For this reason, Xu *et al.* (111) developed the *PrivPC* algorithm to perform CD under DP constraints. Murakonda *et al.* (113) provided a theoretical bound on the error and power of membership inference attacks on an exposed network. Zhang *et al.* (114) showed how to generate synthetic data from a CBN under DP. Rocchi *et al.* (115) used credal networks to mask the released model without degrading its utility, in contrast to DP-based protection techniques. From a causal perspective, privacy protection must be carefully balanced against the need to retain all necessary confounders; failing to collect or to expose key variables due to overly restrictive privacy measures can bias the causal discovery process (62). Further research may shed light on practical privacy leaks in existing methods, clarifying what can and cannot be shared, to better enforce security standards while maintaining the scalability and effectiveness of FCD approaches.

#### 4.2.5 Software availability

The lack of comprehensive software solutions for FCD hinders its development and evaluation on real-world data. Some FCD algorithms have been implemented independently by the respective authors using different programming languages and file formats. Moreover, most implementations require data to be in a tabular format and lack interfaces to learn from CDMs, forcing researchers to use ETL to preprocess CDMs into a tabular form. For instance, Schulz *et al.* (116) conducted a CD study using a single OMOP data set, first ensuring data compatibility with the employed algorithm. The OHDSI community provides the ATLAS and HARES tools for extracting a cohort from an OMOP CDM and populating a data table. At this point, the OHDSI’s ARACHNE system may orchestrate federated epidemiological studies, while the *Vantage6* platform (117) may facilitate more complex FL analyses.

Finally, FCD may incur greater communication and computational overhead than centralised efforts (76). CD is known to be resource-intensive for big data, so most experimentation in the FCD literature is limited to simple CBNs. Future research should thoroughly investigate the computational bottlenecks of FCD and engineer reliable, efficient infrastructures.

## 5 Conclusions

The digitisation of healthcare systems has accelerated the adoption of data-driven, evidence-based medicine. In this context, FCD is emerging as a pivotal paradigm for uncovering causal structures across decentralised data sets without sharing raw data, potentially offering informed RCT designs, improved fairness, and reliable synthetic data generation as presented in Section 4.1. FCD serves as an essential enabler of causal inference, which is equally critical for estimating personalised treatment effects, but depends on first securing a reliable, privacy-preserving causal graph. Individualised care plans require estimating the causal effect of each decision on the patient, often from observational RWE data because randomising those decisions is often unethical or unscalable (62).

FCD is a relatively young field that continues to evolve, and its maturation is best guided by close interaction with clinicians and their domain expertise. Key challenges we described in Section 4.2 include data heterogeneity, population-level biases, the questionable validity of causal assumptions and the lack of quality software implementations. A further critical obstacle is privacy, as discussed in Section 4.2.4: regulations such as GDPR (118) and HIPAA (119) severely restrict the sharing of patient-level data, creating a risk of non-compliance or breach. Federated learning architectures with industry-standard IT security keep raw data local, essentially removing the privacy risks associated with data transfer while enabling collaborative causal discovery (108). Indeed, FL’s privacy-by-design approach aligns with the organisational measures required by GDPR and facilitates compliance with the EU AI Act (120), as discussed in the context of bioinformatics in Malpetti et al. (107). Beyond privacy, federated systems must also address robustness to adversarial attacks, such as membership inference and model inversion, which can originate from both participating parties and external adversaries seeking to extract information about the training data. For settings with the highest risk of patient reidentification, such as rare disease studies, multi-party computation and differential privacy can further strengthen protections. However, multi-party computation incurs substantial computational overhead, and differential privacy may degrade diagnostic or prognostic accuracy. Institutions should adopt these techniques only after a careful risk assessment and a cost-benefit analysis specific to their clinical context.

To translate theoretical methods into safe clinical practice, a structured roadmap is required.

1. **Readiness and common ground.** All institutions should adopt a common data model, such as OMOP, to achieve logical, structural, and vocabulary standardisation (see Section 4.2.1). Local quality checks, semantic harmonisation, and explicit modelling of missing-data mechanisms are mandatory. In parallel, institutions should establish data use agreements, ethics approvals, and federated infrastructure (e.g., secure aggregation nodes). A few successful examples are presented in Section 4.1.1.
2. **Federated causal discovery.** Discovery algorithms execute under appropriate privacy guarantees, with the choice of technique guided by the institutional risk assessment and sensitivity of the data. Because causality formalises knowledge about cause-and-effect relationships and the assumptions we make regarding the data-generating process, prior clinical knowledge must be explicitly encoded as forbidden edges, temporal constraints, or Bayesian priors to guide the search, suppress spurious associations, and make the underlying assumptions transparent (121). The output is a CBN whose validity must be demonstrable. As CBNs are inherently transparent graphical models, they are directly interpretable using classical inference techniques (9, 122), obviating the need for post hoc explainable AI (xAI) methods, which are typically designed for opaque black-box models and can inadvertently capture spurious correlations.
3. **Expert evaluation and iterative refinement.** A multidisciplinary panel should assess the CBN’s plausibility and consistency with a focus on established associations and published evidence. Discrepancies trigger algorithmic refinement and re-evaluation before progressing, a best practice known as a human-in-the-loop model validation (123).
4. **Independent validation and fairness auditing.** The refined CBN undergoes external validation on independent cohorts not used during discovery. A thorough fairness audit should check for structural disparities (for example, missing or inverted edges) across race, gender, socioeconomic status, and other sensitive attributes, ensuring the causal model itself does not encode bias against specific subgroups (see also Section 4.1.3).
5. **Clinical integration and oversight.** The validated CBN is embedded into institutional clinical decision support systems under clear governance (see Section 4.1.1). This integration should enable future causal inference studies to estimate treatment effects without compromising privacy. Continuous monitoring for drift, fairness, and new evidence triggers regular updates.

A fundamental obstacle to realising this roadmap is the absence of standardised evaluation benchmarks and software, as reported in Section 4.2.5. Existing multi-centre clinical trials, with their well-characterised cohorts and known treatment effect estimates, offer a natural starting point for constructing semi-synthetic benchmarks with ground-truth causal structures (55). Federated cancer registries, which already operate across institutional and national boundaries, provide another rich source of real-world distributed data (124). The community must leverage such resources to collaboratively build a benchmark suite that includes a known (real or semi-synthetic) causal ground truth, multiple heterogeneous data sets with controlled distribution shifts and non-random missingness, and realistic privacy constraints. Such a resource would accelerate methodological progress and provide the evidence needed for regulatory acceptance.

By methodically addressing these practical and methodological challenges, FCD can evolve from a research prototype into a mature, safe, and clinically indispensable tool—and, in so doing, unlock the full potential of causal inference across distributed real-world data. The legal and technical landscape will continue to evolve, with legislation such as the EU AI Act progressively shaping how data are shared and used. FL-based approaches are inherently well-positioned to adapt to these developments because they already embed privacy-by-design principles.

## Footnotes

^1^In fact, we may set $U=\underset{k}{⋃}U^{k}$, P(U) its joint distribution, and $P^{k}(U)=\sum_{i\neq k}P(U^{k},U^{i}),\;\forall k$.

^2^The search and inclusion criteria for this review can be found in Supplementary Section S3.
