# Supplementary material for: Federated causal discovery in medicine: trends, opportunities, and challenges
Source: Front Digit Health. 2026 Jul 17;8:1846020. doi: 10.3389/fdgth.2026.1846020 (PMC13424635; doi:10.3389/fdgth.2026.1846020)
Supplement: Supplementary file 1 [file Datasheet1.pdf]

Federated Causal Discovery in Medicine: Supplementary Material

1 BACKGROUND ON CAUSALITY

Identifying cause-and-effect relationships is a fundamental step towards clinical reasoning and the development of tailored strategies that align with broader precision health initiatives. (Glocker et al., 2021) This section provides the rationale and foundations of causal modelling, along with learning techniques, which are also portrayed in Figure 1, main text.

1.1 Causal Machine Learning

Pearl formalised the distinction between probabilistic and causal reasoning through the *ladder of causation*.(Pearl, 2021) The ladder consists of three rungs of increasing complexity and relevance.

1. *Association*: purely about statistical relationships, thus conclusions are based on the patterns and associations in the given observational data. Example: association between changes in biomarker profiles and disease outcomes (e.g., 5-year survival rate in cancer).
2. *Intervention*: studying whether and how interventions impact the system. Example: changes in clinical outcomes in response to a treatment regimen (e.g., cancer recurrence).
3. *Counterfactuals*: Analysing hypothetical scenarios to determine what would have happened under different conditions than those we actually observed. Example: "Would the survival rate change significantly with a different treatment modality?"

Leveraging observational data and expert knowledge to answer research questions at rungs 2 and 3 falls under the broad theme of *causal inference* or *causal machine learning* (CML). (Pearl, 2021) CML aims to discover causal mechanisms to predict which *effects* will be observed under interventions, thus allowing us to answer "What if I make a given decision?" questions (rung 2) and "Why or what if I had acted

differently?" questions (rung 3). Thus, it transcends classical machine learning, which relies on probabilistic associations and can answer only "What is?" questions (rung 1). (Hastie et al., 2009)

The following section introduces a pragmatic and data-driven approach to causation.

1.2 Causal Bayesian Networks

Causal Bayesian Networks (CBNs) provide a pragmatic and rigorous representation of causality, thereby enabling efficient knowledge representation and reasoning at all three rungs. While inferring the cause and effect solely based on *observational* data can be challenging because of *symmetric* relationships, *interventional* data such as those from clinical interventions (e.g., treatment selection) (Brown et al., 2025) or sample selection (e.g., randomisation and inclusion criteria) (Colnet et al., 2024) can provide insight into potential *asymmetry*, hence the direction of the cause-and-effect relationship. (Sachs et al., 2005) A common assumption is that of *causal sufficiency* (Pearl, 2021) which ensures that all the common causes (called *confounders*) are explicitly measured. CBNs also provide a natural representation of confounders between the treatment and the outcome, and can be used to test those assumptions. In RCTs, for instance, the treatment is randomised with respect to a set of factors that might influence both treatment assignment and the outcome, such as patient covariates. As a result, we know that causal effects can only flow from the randomised variables, not the other way around.

A CBN is represented by a pair $(\mathcal{G}, \theta)$, where $\mathcal{G}$ is called *causal graph* (CG) (Pearl, 2021) and θ is a set of parameters associated with $\mathcal{G}$. Each node i in $\mathcal{G}$ corresponds to a random variable X_i, and an edge $X_i \to X_j$ entails that X_i is a *direct cause* of X_j, with changes in X_i directly influencing changes in X_j. The nodes pointing towards X_j are called the *parent set* of X_j and denoted by Π_j. The parameters θ are associated with the joint probability distribution $P(\mathbf{X})$ over all nodes $\mathbf{X} = \{X_i, \ldots, X_n\}$. Each X_i is stochastically independent of its non-descendants given its parents.(Koller and Friedman, 2010) Hence, each node in $\mathcal{G}$ is associated a *local* conditional distribution $P(X_i \mid \Pi_i)$ with parameters θ_i, $\bigcup_i \theta_i = \theta$. The CG $\mathcal{G}$ is often assumed to be a directed and acyclic graph (DAG). This is not a practical restriction: duplicating nodes over different time points and locations makes it trivial to represent both as they unfold in time and space.(Dean and Kanazawa, 1989)

A CBN may be equivalently represented as a *structural causal model* (SCM) mapping the functional relationships between variables. Each $X_i \in \mathbf{X}$'s stochasticity is then expressed by a separate exogenous variable $U_i \in \mathbf{U}$.(Pearl, 2021) The joint distribution

$P(\mathbf{U})$ induces, recursively, the joint $P(\mathbf{X})$ over the measured variables; thus, the set of CBN parameters can be easily derived from $P(\mathbf{U})$ and the functional dependencies.

Definition 1.1 (Structural causal model)**.** An SCM $\mathcal{M}$ consists of a 4-tuple $(\mathbf{U}, \mathbf{X}, \mathcal{F}, P(\mathbf{U}))$ where:

- $\mathbf{U}$ represents a set of exogenous variables determined by factors outside the model.
- $\mathbf{X}$ represents a set $\{X_1, \ldots, X_n\}$ of endogenous variables, determined by other variables in the model, that is, those in $\mathbf{U} \cup \mathbf{X}$.
- $\mathcal{F}$ represents a set of functions $\{f_1, \ldots, f_n\}$ such that each f_i is a mapping from the domain of $U_i \cup \Pi_i$ to X_i, where $U_i \subseteq \mathbf{U}$, $\Pi_i \subseteq \mathbf{X} \setminus \{X_i\}$.

For $i = 1, \ldots, n$, X_i is determined by the structural assignment:

$$X_i \coloneqq f_i(\Pi_i, U_i)\,.$$

This alternative representation is crucial in defining *counterfactuals*, but does not provide any advantage over the standard CBN definition in CD. (Pearl, 2021)

Overall, our ability to perform causal inference is founded on knowing (parts of) the underlying cause-and-effect relationships. These may derive from assumptions, expert opinion, literature research or data and are encoded into the CG. The CG formalises testable implications about conditional (in)dependences between variables: it is not only a descriptive map or a tool for causal inference. As we will discuss below, it provides the language and machinery for articulating assumptions, generating and testing hypotheses, controlling bias in inference, creating synthetic data for exploratory purposes, performing *in silico* experiments, conducting sensitivity analyses for unmeasured confounding, and even planning scenarios for clinical trials.

1.3 Causal Discovery

Causal discovery (CD) (Zanga et al., 2022) consists in learning a CBN $(\mathcal{G}, \theta)$ from the given data while accommodating expert knowledge and established clinical evidence as priors. Figure S1 outlines the typical CD pipeline.

Two broad areas under CD include *structure learning* and *parameter learning*, which enable causal *inference* as a downstream task. While structure learning involves determining the DAG $\mathcal{G}$ that best represents associations in the given multivariate biomedical observational data, parameter learning estimates the marginal conditional probability distributions given the structure $\mathcal{G}$. Inference corresponds to posing queries

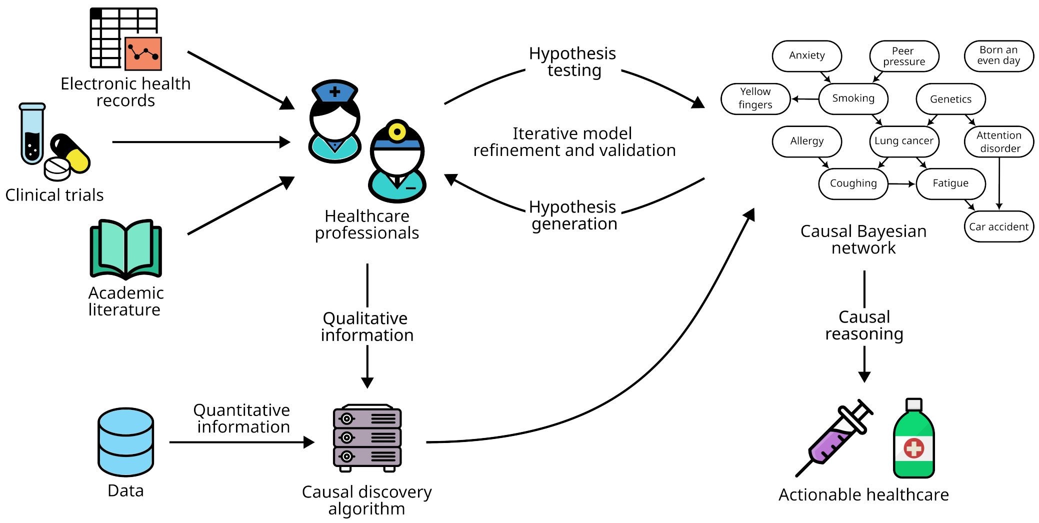

Figure S1. The typical causal discovery pipeline.

to the resulting CBN $(\mathcal{G}, \theta)$. Under rare circumstances, it might be possible to construct $\mathcal{G}$ by leveraging expert knowledge and an extensive literature search. (Xiao-xuan et al., 2007) However, such models have inherent limitations. For instance, *(a)* the retrieved knowledge may be generic and not specific to system under investigation (e.g. molecular signaling mechanism of a tumor subtype); *(b)* the retrieved knowledge may be from diverse hetergeneous sources challenging its integration (e.g. cell lines, tissues, species);*(c)* the retrieved knowledge may not be representative of the population or demographics of interest (e.g. variations in social determinants); *(d)* the system under investigationg may be novel with minimal precedence or prior knowledge (e.g. COVID-19). These challenges implicitly demand modelling the CBN $\mathcal{G}$ and its parameters θ in a data-driven $(\mathcal{D})$ and evidence-based manner.

To this end, CD is posed as a Bayesian optimisation problem comprising structure and parameter learning:

$$\underbrace{P(\mathcal{G}, \theta \mid \mathcal{D})}_{\substack{\text{CBN} \\ \text{learning}}} = \underbrace{P(\mathcal{G} \mid \mathcal{D})}_{\substack{\text{Structure} \\ \text{learning}}} \cdot \underbrace{P(\theta \mid \mathcal{G}, \mathcal{D})}_{\substack{\text{Parameter} \\ \text{learning}}}.$$

As mentioned in Section 1.2, the DAG $\mathcal{G}$ may not be uniquely identifiable when relying solely on observational data, resulting in probabilistically indistinguishable structures, also referred to as the *Markov equivalence class* of $\mathcal{G}$.(Koller and

Friedman, 2010) Examples include network *motifs* or prevalent structures such as *chains* $X_i \to X_j \to X_k$, $X_i \leftarrow X_j \leftarrow X_k$, and *fork* $X_i \leftarrow X_j \to X_k$. The corresponding Markov equivalence class is given by the undirected graph $X_i - X_j - X_k$ since chains and forks are probabilistically indistinguishable. Therefore, a CBN is represented as *completed partially DAG* (CPDAG), typically comprising directed as well as undirected edges. An edge in a CPDAG is directed if and only if it is directed across all DAGs in the Markov equivalence class. However, interventional data can help identify the direction of undirected edges in the CPDAG.(Sachs et al., 2005) Several factors, such as the structure learning algorithm, sample size, dimensionality, and distributional assumptions of the multivariate data, can also impact identifying the direction of the edges. (Zanga et al., 2022)

Structure learning is a hard computational problem because the number of possible DAG structures increases super-exponentially with dimensionality. (Bouckaert, 1995) Algorithms to tackle it broadly comprise *constraint-based*, *score-based*, and *hybrid approaches*. Score-based approaches that identify the DAG that best represents the given data using a search criterion in conjunction with a scoring function, such as LiNGAM and NOTEARS, have been used successfully to determine edge directions. LiNGAM assumes that the distribution of exogenous variables is such that different edge directions imply different likelihood values, allowing for disambiguation. Differentiable algorithms like NOTEARS leverage scale differences between the exogenous variables and add penalty terms to the likelihood to ensure $\mathcal{G}$ is acyclic. In contrast, constraint-based approaches recover the optimal DAG using tests for conditional independence and are implicitly limited in determining edge directions by the symmetry of the relationships. Hybrid approaches combine constraint-based and score-based algorithms to identify the optimal DAG. The performance of these approaches and their computational complexity vary considerably.(Scutari et al., 2019) As noted earlier, once $\mathcal{G}$ is determined, the associated parameters can be learned from data and domain knowledge using *maximum likelihood* (MLE) and *Bayesian posterior* estimators.(Koller and Friedman, 2010)

While expert knowledge may not be enough to construct $\mathcal{G}$ by itself, it may assist in constraining the search space of candidate DAGs. These constraints in turn can be either *soft* or *hard*. (Constantinou et al., 2023) Soft constraints help guide the structure learning algorithm; *hard* constraints explicitly eliminate specific structures, minimising the search space of the structure learning process.

2 FEDERATED LEARNING

Federated learning (FL) trains a model locally via global updates without explicit sharing of *protected health information* across healthcare organisations, thereby complying with data protection regulations. (McMahan et al., 2017) Recent studies show how FL improves breast density classification models (accuracy up by 6%, generalizability up by 46%),(Roth et al., 2020) COVID-19 outcome prediction at both 24h and 72h (up 16% and 38%)(Dayan et al., 2021) and rare tumour segmentation (up by 23–33% and 15%)(Pati et al., 2022) compared to single-organisation analyses. Early-stage applications that build predictive models from electronic health records (Brisimi et al., 2018) have also confirmed no practical performance degradation compared with centralised analysis. Xu *et al.*(Xu et al., 2021) provides an excellent introduction to FL in healthcare.

FL architectures typically follow a *client-server* design, in which models are trained locally on data at individual clients and then updated globally. (Zhang et al., 2021) A taxonomy of FL along its key aspects (*data partitioning*, *architecture*, *algorithms*) is shown in Figure 1 in the main paper. *Data partitioning* describes how data are split across clients (see Figure 3 in the main paper). The *architecture* defines the roles of clients and servers and how they exchange information. Finally, the FL *algorithm* defines how models are learned and what privacy-preserving techniques are adopted.

3 REVIEW INCLUSION CRITERIA

The FCD algorithms reviewed in this manuscript were identified through a targeted search of three major databases: IEEE Xplore, ACM Digital Library, and Google Scholar. Moreover, we complemented them by manual screening of the proceedings of key venues (such as AISTATS, AAAI, ICLR, IJCAI, NeurIPS, and ECML-PKDD) and their co-located workshops, including the KDD Workshop on Causal Discovery. Searches were conducted using combinations of the terms “federated”, “structure”, “learning”, “causal”, “Bayesian”, “network“, ”discovery” and “structure learning”. Examples of queries include “federated causal discovery” and “federated Bayesian structure learning”. The considered time span was 10 years, with the last search performed in September 2025. Preprints hosted on arXiv were considered eligible and included when no peer-reviewed version was available at the time of the search. In particular, an FCD algorithm has to: (i) explicitly frame itself as a federated learning approach according to the taxonomy in Table 2, meaning that raw data are never shared and aggregation takes place at a central server; (ii) target the

structure learning problem, as opposed to causal inference that assumes a known DAG; (iii) provide sufficient methodological detail to characterise the algorithm along the dimensions reported in Table 3. Methods addressing only parameter estimation given a fixed graph, or federated predictive modelling without an explicit structural learning component, were excluded.

REFERENCES

Glocker B, Musolesi M, Richens J, Uhler C. Causality in Digital Medicine. *Nature Communications* **12** (2021) 5471.

Pearl J. *Causal Inference in Statistics* (Wiley) (2021).

Hastie T, Tibshirani R, Friedman J. *The Elements of Statistical Learning: Data Mining, Inference, and Prediction* (Springer), 2nd edn. (2009).

Brown BC, Tokolyi A, Morris JA, Lappalainen T, Knowles DA. Large-Scale Causal Discovery Using Interventional Data Sheds Light on Gene Network Structure in k562 Cells. *Nature Communications* **16** (2025). doi:10.1038/s41467-025-64353-7.

Colnet B, Mayer I, Chen G, Dieng A, Li R, Varoquaux G, et al. Causal Inference Methods for Combining Randomized Trials and Observational Studies: A Review. *Statistical Science* **39** (2024) 165–191. doi:10.1214/23-sts889.

Sachs K, Perez O, Pe'er D, Lauffenburger DA, Nolan GP. Causal Protein-Signaling Networks Derived From Multiparameter Single-Cell Data. *Science* **308** (2005) 523–529. doi:10.1126/science.1105809.

Koller D, Friedman N. *Probabilistic Graphical Models* (MIT Press) (2010).

Dean T, Kanazawa K. A Model for Reasoning About Persistence and Causation. *Computational Intelligence* **5** (1989) 142–150. doi:10.1111/j.1467-8640.1989.tb00324.x.

Zanga A, Ozkirimli E, Stella F. A Survey on Causal Discovery: Theory and Practice. *International Journal of Approximate Reasoning* **151** (2022) 101–129. doi:10.1016/j.ijar.2022.09.004.

Xiao-xuan H, Hui W, Shuo W. Using Expert's Knowledge to Build Bayesian Networks. *2007 International Conference on Computational Intelligence and Security Workshops (CISW 2007)* (2007), 220–223. doi:10.1109/cisw.2007.4425484.

Bouckaert RR. *Bayesian Belief Networks: From Construction to Inference*. Ph.D. thesis, Utrecht University, The Netherlands (1995).

Scutari M, Graafland CE, Gutiérrez JM. Who Learns Better Bayesian Network Structures: Accuracy and Speed of Structure Learning Algorithms. *International*

Journal of Approximate Reasoning **115** (2019) 235–253. doi:10.1016/j.ijar.2019.10.003.

Constantinou AC, Guo Z, Kitson NK. The Impact of Prior Knowledge on Causal Structure Learning. *Knowledge Information Systems* **65** (2023) 3385–3434. doi:10.1007/s10115-023-01858-x.

McMahan B, Moore E, Ramage D, Hampson S, y Arcas BA. Communication-Efficient Learning of Deep Networks From Decentralized Data. *Proceedings of Machine Learning Research* **54 (AISTATS)** (2017) 1273–1282.

Roth HR, Chang K, Singh P, Neumark N, Li W, Gupta V, et al. Federated Learning for Breast Density Classification: A Real-World Implementation. *Domain Adaptation and Representation Transfer, and Distributed and Collaborative Learning: 2nd MICCAI Workshop, DART 2020, and 1st MICCAI Workshop, DCL 2020* (2020), 181–191. doi:10.1007/978-3-030-60548-3_18.

Dayan I, Roth HR, Zhong A, Harouni A, Gentili A, Abidin AZ, et al. Federated Learning for Predicting Clinical Outcomes in Patients with COVID-19. *Nature Medicine* **27** (2021) 1735–1743. doi:10.1038/s41591-021-01506-3.

Pati S, Baid U, Edwards B, Sheller M, Wang S, Reina GA, et al. Federated Learning Enables Big Data for Rare Cancer Boundary Detection. *Nature Communications* **13** (2022) 7346. doi:10.1038/s41467-022-33407-5.

Brisimi TS, Chen R, Mela T, Olshevsky A, Paschalidis IC, Shi W. Federated Learning of Predictive Models From Federated Electronic Health Records. *International Journal of Medical Informatics* **112** (2018) 59–67. doi:10.1016/j.ijmedinf.2018.01.007.

Xu J, Glicksberg BS, Su C, Walker P, Bian J, Wang F. Federated Learning for Healthcare Informatics. *Journal of Healthcare Informatics Research* **5** (2021) 1–19.

Zhang C, Xie Y, Bai H, Yu B, Li W, Gao Y. A Survey on Federated Learning. *Knowledge-Based Systems* **216** (2021) 106775. doi:10.1016/j.knosys.2021.106775.
